# "HOW WILL I LIVE THIS LIFE I AM TRYING TO SAVE?": EXPLORING DUALITIES IN CANCER SURVIVORSHIP EXPERIENCES IN NIGERIA

**DOI:** 10.1093/geroni/igac059.2242

**Published:** 2022-12-20

**Authors:** Candidus Nwakasi, Darlingtina Esiaka, Runcie Chidebe, Khadijat Banwo Fatai, Gloria Orji

**Affiliations:** Providence College, Providence, Rhode Island, United States; Robert Wood Johnson Medical School, Rutgers University, Newark, New Jersey, United States; Health and Psychological Trust Center, Abuja, Federal Capital Territory, Nigeria; Project PinkBlue, Abuja, Federal Capital Territory, Nigeria; Network of People Impacted by Cancer in Nigera, Abuja, Federal Capital Territory, Nigeria

## Abstract

Objectives. Cancer mortality rate is high in Nigeria with about 102,000 new cases and 72,000 deaths per year. Cancer incidence rate is projected to increase with its rapidly aging population and growth — emphasizing its growing cancer burden. Due to Nigeria’s weak health system, limited cancer therapy and expert oncological services, lack of cancer education and awareness, and sub-optimal implementation of its national cancer control plan, there is an increase in the risk of poor outcomes from cancer. To further understand the mechanism behind the poor outcomes, we explored cancer survivorship experiences of breast cancer survivors in Nigeria. Methods. The study employed a qualitative descriptive method. Semi-structured interviews were conducted with a purposive sample of 30 female breast cancer survivors in Abuja, Nigeria (Mage = 42 years). Their responses were transcribed, coded, and analyzed for themes. Results. The four major themes identified were: 1) life after cancer diagnosis (hope vs despair), 2) faith and religion (coping with faith vs issues with religion), 3) accessing cancer therapy (alternative therapy vs orthodox therapy), and 4) relationship with medical providers (encouragement from medical providers vs hinderance from medical providers). Discussion. Cancer death rates may be high but there is also a growing number of survivors in Nigeria. It is crucial to improve the health and well-being of Nigerians from the time of diagnosis until end of life, thus, the focus of cancer survivorship. The findings point to the critical need for policies to help strengthen cancer survivorship in Nigeria.

